# Mechanical Properties and Microstructure of Epoxy Mortars Made with Polyethylene and Poly(Ethylene Terephthalate) Waste

**DOI:** 10.3390/ma14092203

**Published:** 2021-04-25

**Authors:** Bernardeta Dębska, Guilherme Jorge Brigolini Silva

**Affiliations:** 1 Department of Building Engineering, Rzeszow University of Technology, ul. Poznańska 2, 35-959 Rzeszów, Poland; 2 Departamento de Engenharia Civil, Universidade Federal de Ouro Preto, Campus Morro do Cruzeiro, Ouro Preto CEP 35.400.000, Brazil; guilhermebrigolini@ufop.edu.br

**Keywords:** epoxy resin, PET waste, PE waste, design of experiment, profiles of approximated, composite microstructure

## Abstract

The article describes the results of a study to determine the simultaneous effect of polyethylene terephthalate waste (PET) and polyethylene (PE) on the strength characteristics and bulk density of epoxy mortars. In these mortars, 9 wt.% of the polymer binder was replaced by glycolysate which was made from PET waste and propylene glycol. Additionally, 0–10 vol.% of the aggregate was substituted with PE agglomerate made from plastic bags waste, respectively. The modification of the composition of epoxy mortar has a special environmental and economic aspect. It also allows to protect natural sources of the aggregate, while reducing the amount of waste and reducing problems arising from the need to store them. The resulting composite has very good strength properties. With the substitution of 9 wt.% of resin and 5 vol.% of sand, a flexural strength of 35.7 MPa and a compressive strength of 101.1 MPa was obtained. The results of the microstructure study of the obtained mortars constitute a significant part of the paper.

## 1. Introduction

**Figure 1:**
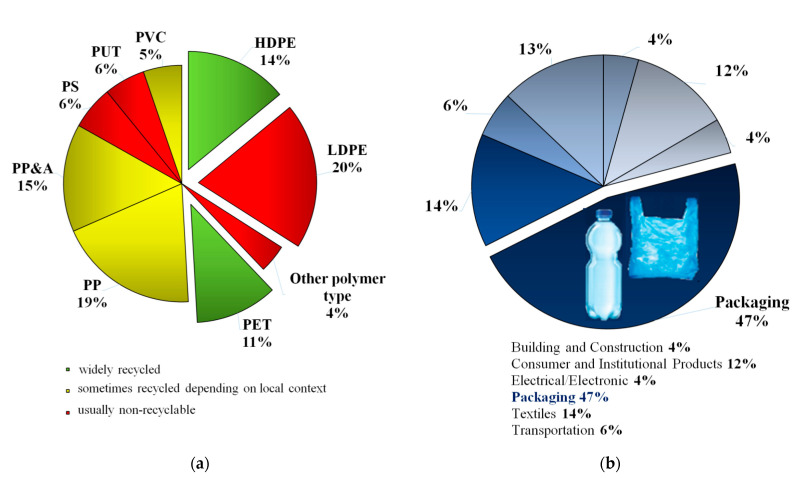
Plastic waste in the world (2015 production): (**a**) by polymer type, (**b**) by sectors.

The 21st century is characterized by continuous growth in production and consumption. This behavior consumes the earth’s resources, which are after all limited. In addition, it leads to the extinction of species and the pollution of the atmosphere and oceans with huge amounts of waste, mainly plastic [1,2]. The scale of the problem is well illustrated by the fact that billions of plastic objects have formed an artificial island in the Pacific Ocean. Traditional plastic does not break down into natural substances. If left in the ocean, it will decompose into microscopic fragments over years under the influence of seawater and sunlight, increasing its toxicity [3], as microplastics can also facilitate the transfer of toxic chemicals and pathogens. Despite these undeniable risks, since the 1960s, global plastic production has increased 20-fold, reaching 322 million tons in 2015. This number is projected to further double in the next twenty years [4]. If this trend continues then by 2050 plastic could account for 15% of greenhouse gas emissions and there could be more plastic in the sea than fish [5]. Among the plastic waste generated globally, polyolefins (polyethylene (PE) and polypropylene (PP)) and polyethylene terephthalate (PET) have the largest contribution, as can be seen in Figure 1a. A significant amount of this waste comprises packaging materials such as PE bags and sacks or PET bottles and packaging, which are very difficult to dispose of [6,7]. More than 40% of plastic waste comes from packaging (Figure 1b), but only 40% of this packaging is recycled [5,8]. Climate change experts show that there will be no future for humanity in a few decades or by the end of this century as it continues to pursue growth while generating high emissions. Solutions to this problem include converting infrastructure to low-carbon, adopting a different concept of transportation, reducing long-distance trade, and thereby strengthening local production. Long-distance transport has also been reduced in recent months by the outbreak of the COVID-19 epidemic.

It is therefore advisable to look for waste applications that will enable the management of these troublesome materials at the place of their original use, but at the same time reduce the consumption and transportation costs of local natural resources. Such waste applications include the substitution of natural aggregate in concretes and mortars [9,10,11,12,13,14,15]. For example, if a concrete mixture consists of approximately 30% sand, when 10% of this aggregate is replaced by plastic waste and assuming an annual production of approximately 20 billion tons, approximately 800 million tons of sand could be saved per year [16]. Researches in this field, carried out in many scientific centers in the world, are highly recommended, because they lead to obtaining concretes with various properties, depending on many features of the waste material, including the type of plastic, size and shape of particles, degree of substitution [17,18]. At the same time, at this point it is worth pointing out that not every application of concrete elements is a structural one. There are also such examples of applications where strength is not the main criterion, and what matters is, for example, the weight of the element, chemical resistance, adhesion to other materials. Concrete and resin mortars seem to be particularly attractive in terms of waste disposal. They are composed predominantly of aggregate (up to 90%), as well as synthetic resin and its hardener and/or additives or admixtures [19,20]. They have found a special place among construction materials in applications such as industrial flooring, production of prefabricated elements including bridge and road drainage elements, artificial marbles, machine foundations, repair systems, among others. These composites are characterized by very high strength parameters, with chemical and corrosion resistance, in addition to low water absorption, vibration damping capacity, thermal stability, and very short time to achieve operational efficiency [21,22,23]. Due to the great popularity of cement concretes, there are many more examples in the literature describing the processes of modification of these composites with plastic waste (including: PE and PET) [24,25,26,27,28] than resin concretes. Resin composites containing plastic waste were studied by Reis et al. [29,30], Vidales et al. [31], Dębska and Lichołai [21], among others. Reis et al. studied epoxy and polyester mortars in which 0–20% sand was replaced by washed and shredded PET waste. The strength parameters of these composites deteriorated as the waste content increased, but at the same time a lighter and more ductile product was obtained, exhibiting less brittle damage, which was confirmed by fracture mechanics results. Vidales and his team obtained mortars based on synthetized polyester resin using PET waste chemically processed by glycolysis and PET particles. These authors noted that increasing the resin content in the composite provided better sand wettability and improved the connection between phases [31]. Dębska and Licholai observed an increase in the strength parameters of epoxy mortars containing glycolysates formed from PET waste [21].

This article deals with epoxy mortars modified in two ways. First, 9 wt.% of the epoxy binder was replaced by a glycolysate based on propylene glycol and waste poly(ethylene terephthalate) (PET). Additionally, sand was partially (0–10 vol.%) replaced by a polyethylene (PE) agglomerate obtained by processing waste plastic bags. The proposed material solution is important for both environmental and economic reasons. It also positively influences the flexural strength of epoxy mortars, which is at the level of 30.3–35.7 MPa. A material with excellent compressive strength, varying in the range of 86.0–107.2 MPa, was obtained. The use of PE waste allowed also to reduce the weight of the product. The use of the experimental design methods in the conducted studies facilitated the determination of the most advantageous composition of mortars with respect to the determined properties. It also resulted in a significant reduction in the number of samples of the tested composites, which allowed to shorten the time and decrease the costs of the conducted experiments. Microstructural studies made it possible to correlate the strength test results with the structure of the mortars studied.

## 2. Materials and Methods

### 2.1. Materials

Epoxy resin (based on bisphenol A) (Epidian 5, CIECH Sarzyna S.A.) was the binder in the composite samples made. The resin was characterized by molecular weight 450 g/mol and epoxy number LE = 0.49 mol/100 g, density of 1.17 g/cm^3^ and viscosity of 30,000 mPa∙s.

**Figure 2:**
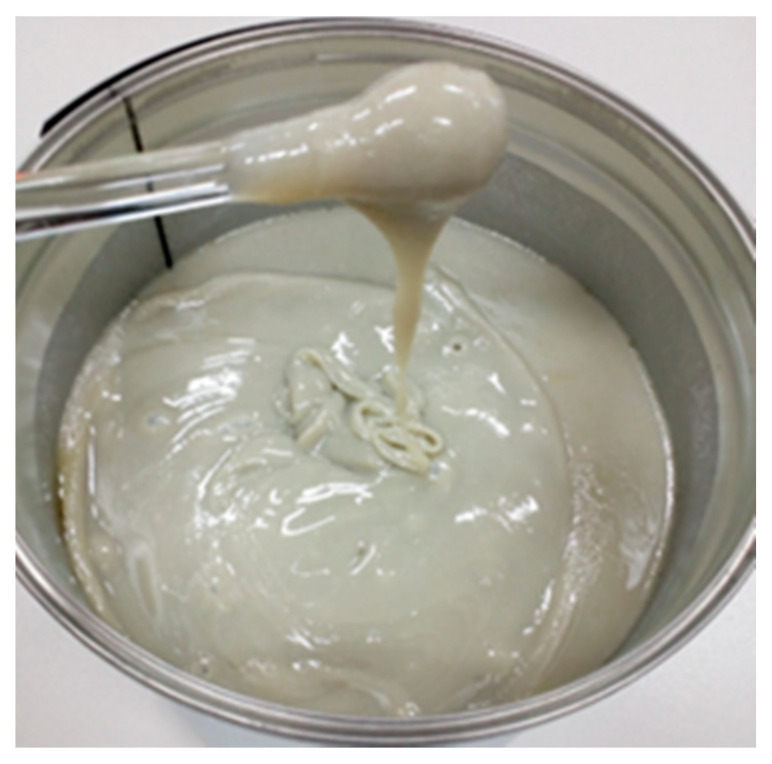
Glycolisate based on waste PET.

The resin was partially replaced (9 wt.%) with glycolysate based on propylene glycol and waste poly(ethylene terephthalate) from waste beverage bottles. The process of glycolysis was carried out at 210 °C in the presence of zinc acetate as a catalyst, at an assumed PET/glycol molar ratio of 1/1.7. The glycolysate was in the form of milky-gray semifluid wax (Figure 2).

Z-1 hardener was used in an amount of 10 wt.% of the resin weight. The main component of the hardener was triethylenetetramine. The hardener was in the form of light yellow liquid with density of 0.981 g/cm^3^ and viscosity in the range of 20–30 mPa∙s.

The quartz sand, which density is 2.65 g/cm^3^, acted as the aggregate.

**Figure 3:**
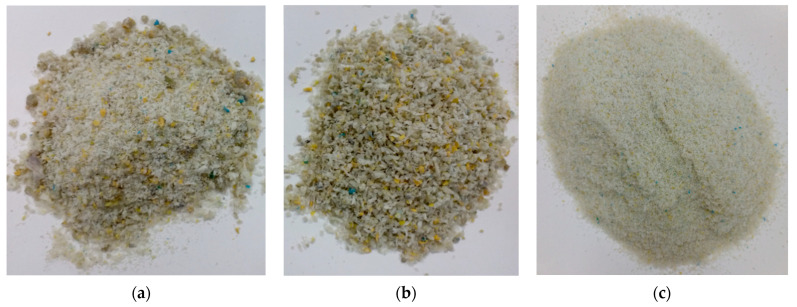
PE agglomerate: (**a**) before the selection into fractions, (**b**) 1 mm fraction, (**c**) 0.25 mm fraction.

A partial substitute for sand was an agglomerate PE waste, made from waste plastic bags, made available by a local company that produces this type of agglomerate. Nowadays, mechanical methods are much more frequently used in foil recycling. Depending on the level of the contamination of the plastic bags, the recycling processes vary. The agglomerate used in the study came from contaminated foils, which were subjected to cyclic agglomeration with separate washing and drying processes of the shredded foil. In the process of agglomerate preparation, the plastic packaging obtained from selective waste collection was ground to produce flakes, cleaned in separation baths and passed through a system of vibrating chutes to drain water. In the next stage carried out at increased temperature (ranging from 150 °C to 170 °C), as a result of the compacting process, the flakes were transformed into an agglomerate, which is a granular form of plastic of irregular shape and varied grain size. Figure 3 shows pictures of different fractions of PE agglomerate.

### 2.2. Methods

The epoxy binder was thoroughly mixed with the glycolysate until a homogeneous mixture was obtained. The composition prepared in this way was annealed for 60 min at 85 °C to allow the reaction between the epoxy groups of the resin and the hydroxyl groups of the PET glycolysate. After cooling to ambient temperature, a hardener at 10% in proportion to the weight of the resin was added to the mixture. The components of the mortar were mixed in a laboratory mixer at 140 ± 5 rpm for 3 min. The mortar prepared in this way was molded into steel molds, making it possible to obtain three samples with dimensions of 40 × 40 × 160 mm^3^. For filling one mold of mortar not modified with PE waste, 1620 g of sand and 414.85 g of resin composition (Epidian 5 epoxy resin + PET glycolysate) were used. For subsequent compositions, corresponding to each point of the experimental plan, sand was replaced by volume with PE waste and the amount of resin composition with glycolysate was modified according to the data in Table 1. Additionally, control samples were made which did not contain any type of waste. The composition of such mortar required to fill one mold for strength testing is: 1620 g of standard sand, 414.85 g of epoxy resin, and 41.5 g of the hardener. Three samples from each series (for each point of the experimental plan) were made. The samples were seasoned under laboratory conditions for 7 days.

The following strength machines were used to determine the flexural and compressive strength: Cometech Testing Machines Co., Taichung, Taiwan and MATEST S.p.A., Arcore, Italy. Special inserts for testing machines were used according to PN-EN 196-1 [32].

The bulk density of epoxy mortars was calculated as the ratio of the dry mass to the total volume.

A laboratory shaker with a set of control sieves (LPzE-3e) was used to develop the aggregate particle size distribution.

The PE waste was crushed until it passed through a 200 mesh (75 micron) sieve and evaluated by dispersive energy X-ray fluorescence (XRF) in Epsilon-3x-PANalitic equip-ment and X-ray diffraction (XRD) using Bruker D2 Radiation Phaser-CuKa (k = 1.54184 Å) with a Ni filter.

### 2.3. Central Compositional Plan Methodology

Conducting destructive tests involves the need to make a significant number of samples. In order to reduce their number, but at the same time obtain full scientific information regarding the tested mortars, the experiment theory was applied. Following the previous own research [33], a compositional master plan with a response surface was selected. To plan the experiment and to perform subsequent analyses, the Statistica 12 program (StatSoft Inc., Kraków) [34] were used. The composition of the designed mortars was characterized by two input variables:

- x_1_—waste PE content (% PE)—took values from 0 to 10 vol.% and was a substitute for sand;
- x_2_—the volume ratio of resin to aggregate R/A with values in the range of 0.33–0.82.

The experiment plan consisted of 10 points. It was assumed that the focal point study would be repeated (plan points 6 and 7 do not differ in composition). Data characterizing each point in the plan are provided in Table 1.

For the input variables (z) (bending and compressive strength as well as bulk density), approximating functions (response surfaces) were determined. It was assumed that the approximation function has the form of a second degree polynomial (1). It describes the relationship between output and input quantities.

(1) $$\hat{z}=A_{0}+A_{1}x_{1}+A_{2}x_{1}^{2}+A_{3}x_{2}+A_{4}x_{2}^{2}+A_{5}x_{1}x_{2}$$

where:

*ẑ*—value of the test object function for real variable values,
*x*_1_—percentage share of waste PE (% PE),
*x*_2_—resin to aggregate ratio (R/A),
*A_i_*—coefficients of the equation for real variables.

At the end of the research, such a mortar composition was determined that allows obtaining a composite characterized not only by the most favorable values of strength parameters, but also by low volumetric density. For this purpose the utility profile tab of the Statistica program’s was applied, allowing the approximate values of the three tested outputs to be converted into a single value of the total utility of the tested composites.

## 3. Results and Discussion

**Figure 4:**
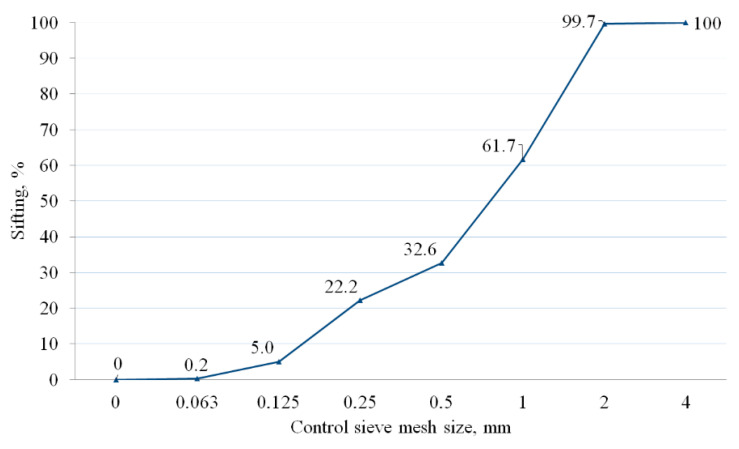
Sand grain size distribution curve.

Sand grain size distribution curve is shown in Figure 4. In accordance with the distribution shown in Figure 4, individual sand fractions were replaced in volume with PE waste.

**Figure 5:**
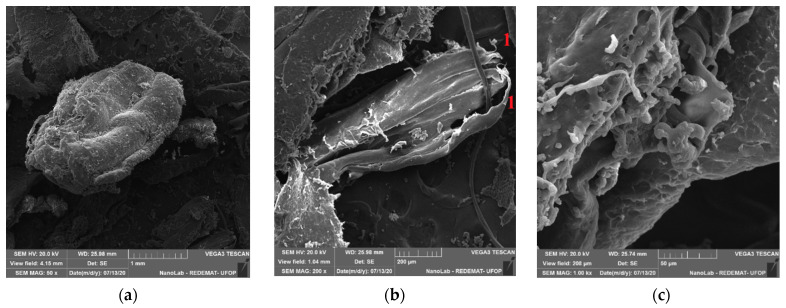
SEM images of waste PE at 50× (**a**), 200× (**b**), and 1000× (**c**) magnifications.

The SEM-SE images of the PE waste are presented in Figure 5.

It can be seen in Figure 5b at the ends of the particle (point 1) an elongated shape, possibly obtained after the process of cutting the polyethylene bags.

Table 2 shows the chemical composition of the quartz sand.

The elementary analysis of the polyethylene waste identified high levels of Ca, Ti; average levels of Al, Si, P, S, Cl, Zn, Fe; low levels of K, Mg, V, Cr, As, and Pb. The high presence of the Ca element is due to the frequent use of filling additives such as CaCO_3_. The amount of CaCO_3_ added to the PE can influence its mechanical properties, such as fracture tensile strength. The tensile modulus of the PE compound increases with the addition of CaCO_3_ [35]. Therefore, PE with different CaCO_3_ contents influences the mechanical properties of the epoxy-PE compound.

**Figure 6:**
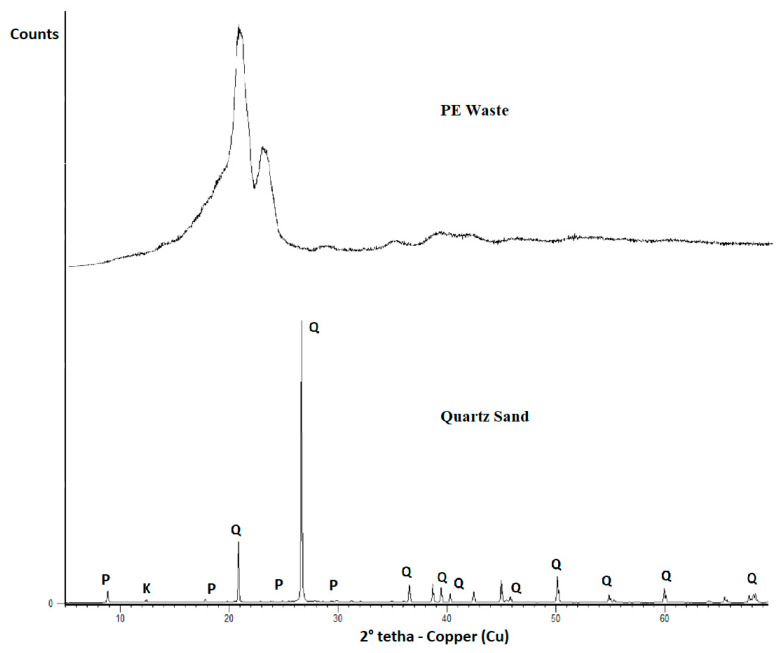
XRD of the PE waste and quartz sand.

The XRD patterns of PE waste and quartz sand are presented in Figure 6. The diffractogram of PE waste is in agreement with other results found in the literature [36,37,38]. The XRD pattern of PE showed the two peaks at Bragg angles 2θ = 20.9° and 23.1°, characteristic of low density polyethylene and a semi crystalline material.

### 3.1. Flexural Strength

**Figure 7:**
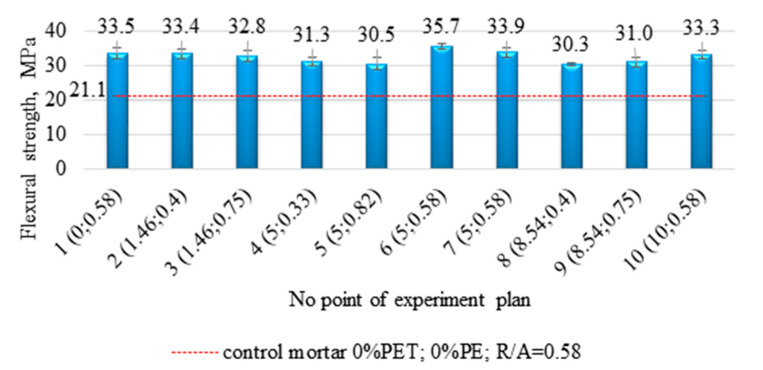
The compilation of the mean values of the flexural strength at different points of the experimental plan.

The average flexural strength values measured for the samples corresponding to each point of the experimental plan are summarized in Figure 7. The standard deviations are also marked. The values of the determined strength for all compositions are at a very high level exceeding 30 MPa. The lowest value of 30.3 MPa was recorded for the mortar containing 8.54% of waste PE at R/A equal to 0.4 (the 8th point of the plan), while the highest value of 35.7 MPa characterized the 6th point of the experimental plan (%PE = 5%, R/A = 0.58). In addition, the horizontal line indicates the flexural strength value obtained for epoxy mortars without PET glycolysate and PE waste, characterized by a resin to aggregate ratio of 0.58.

**Figure 8:**
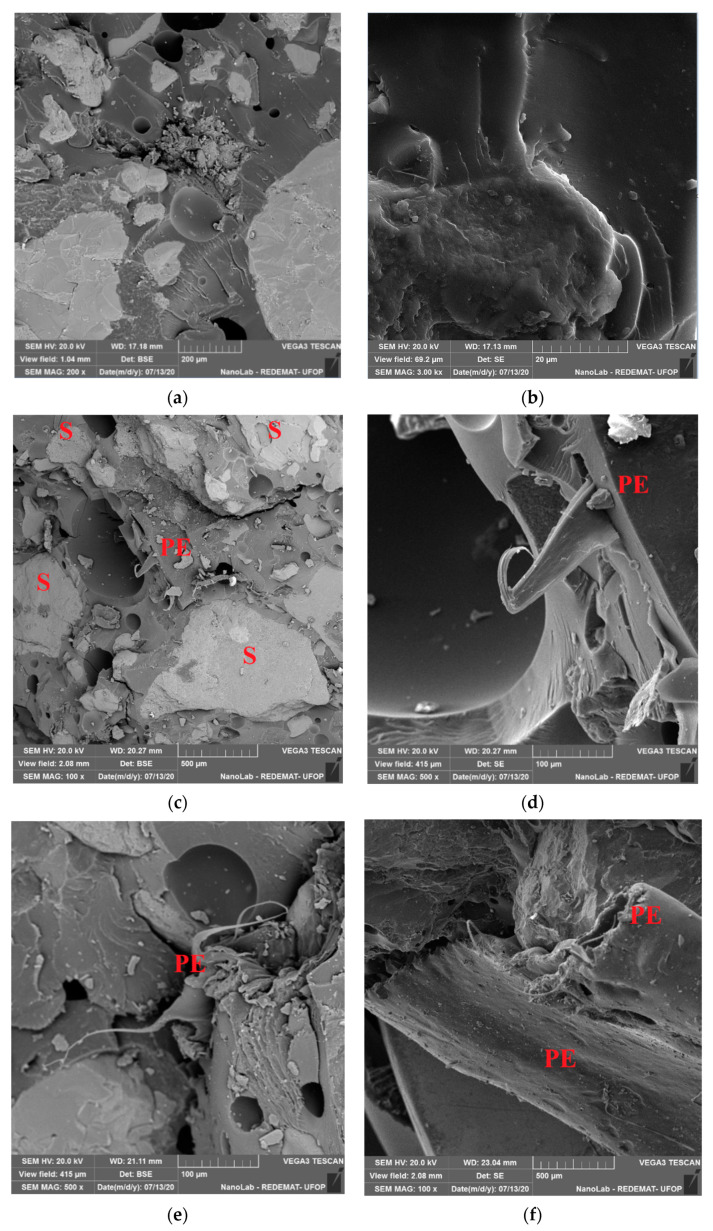
Backscattered electron (BSE) and Secondary electron (SE) images of mortar samples marked in the experiment plan as: 1 (BSE—(**a**), SE—(**b**)), 10 (BSE—(**c**,**e**) ; SE—(**d**,**f**)), PE—polyethylene waste, S—quartz sand.

**Figure 9:**
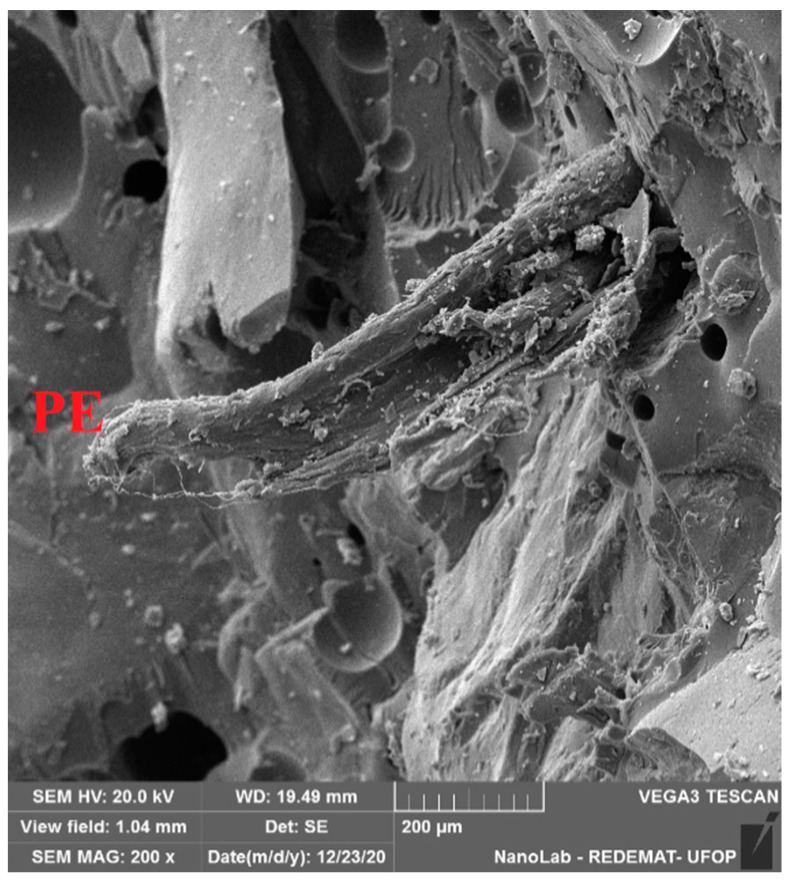
SEM-SE micrographs of the Composition 5—PE: PE waste.

The proposed modification allows for improving the flexural strength by more than 10 MPa (more than 50%). Comparing the strength values of the test mortar with the mortar containing additionally PET glycolysate (the first point of the experimental plan) one can confirm the conclusions described in earlier publications [21]. The substitution of the part of the resin with PET glycolysate causes a significant increase (in this case by 12.4 MPa) of flexural strength of epoxy mortars. SEM images taken for the mortar with the composition assigned for the first point of the experimental plan (Figure 8a,b) show a good bond between the resin and PET glycolysate, the matrix is smooth and homogeneous. Further, the strength results obtained for compositions 1, 6 and 7 and 10, i.e., with the same R/A = 0.58, were compared with the test mortar. Moreover, the PE waste (even at 10% sand replacement rate) has a favorable effect on the studied mechanical property. The flexural strength values obtained can be explained by the phenomenon of the occurrence of interactions between the waste particles and the polymer matrix, as shown in the paper by Martínez-López and the team [39]. Such interactions depend on the morphology of both the resin and the waste particles. Images of the PE waste taken with a scanning microscope (Figure 5) show that the surface of the PE waste is not smooth. The surface roughness observed for the PE particles facilitates mechanical anchoring between the polymer matrix and the PE particles and consequently an increase in flexural strength. Microphotographs (Figure 8c–f) show waste particles that can have a fiber-like effect. Figure 9 shows a crack region in the composition 5 where it is possible to see a PE particle that may be behaving like a fiber. With a higher proportion of waste and a lower resin to aggregate ratio, the coating of the aggregate particles by the resin may be impeded, resulting in some voids that may provide a place for crack initiation during mechanical tests.

**Figure 10:**
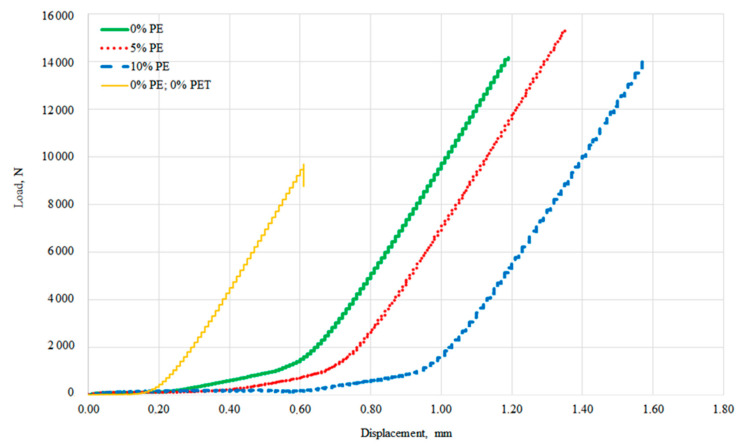
Load vs. displacement curves for flexural strength.

As it can be observed in Figure 10, the addition of glycolysate and PE waste significantly improves the plastic properties of mortars compared to the control samples. However, with a higher degree of sand substitution with PE waste agglomerate (10 vol.%), the plasticity improvement is already at the expense of strength reduction.

**Figure 11:**
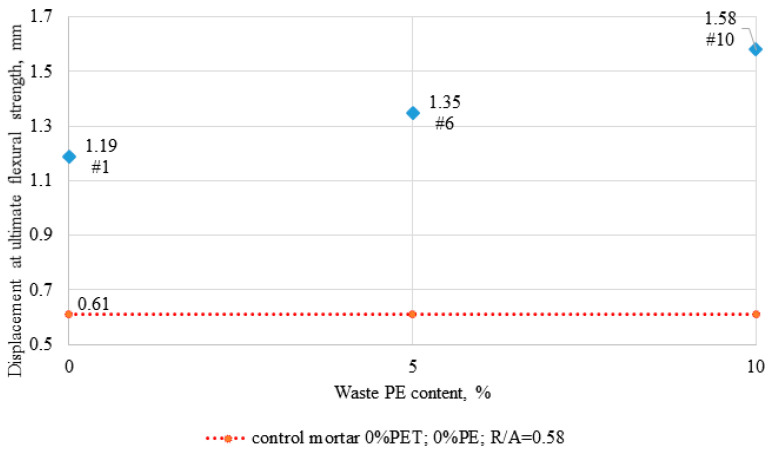
Displacement at ultimate flexural strength depending on the PE waste content, designated at experiment plan points numbered 1, 6, and 10.

The plasticity was evaluated by displacement testing at maximum load and the results are shown in Figure 11. It reveals a significant improvement in the flexural ductility of epoxy composites as the degree of sand substitution with PE waste increases. Both types of modifiers improve the ductility and lead to less brittle failure of mortars. In Figure 10 for the control sample, the curve is clearly terminated, while the other curves behave differently, which is due to the fact that even when the scratching occurs and the maximum force is recorded, the samples do not disintegrate, but remain somehow connected by the PE waste particles. A similar behavior of the samples was observed in the study performed by Martínez-López and the team [39] for polyester mortars containing PET waste such as granules, tire rubber, and polycarbonate, and in the study of Ribeiro et al. [40]. Studies by Reis and Carneiro also confirmed that shredded PET waste particles contribute to a significant increase in the elastic modulus of epoxy mortars [29]. However, these authors did not achieve such high flexural strength values as described in this paper. Also, the displacement at ultimate flexural strength obtained by our team is much higher (1.19–1.58 mm) than that reported by Martínez-López and the team (0.83–0.89 mm).

Using the experiment planning method made it possible to find a response surface function that matched the results of the flexural strength measurements. In this case, the function with the general Formula (1) took the form of (2):

f_f_ = 14.08 − 0.06(%PE) + 73.55(R/A) − 67.01(R/A)^2^ (2)

**Figure 12:**
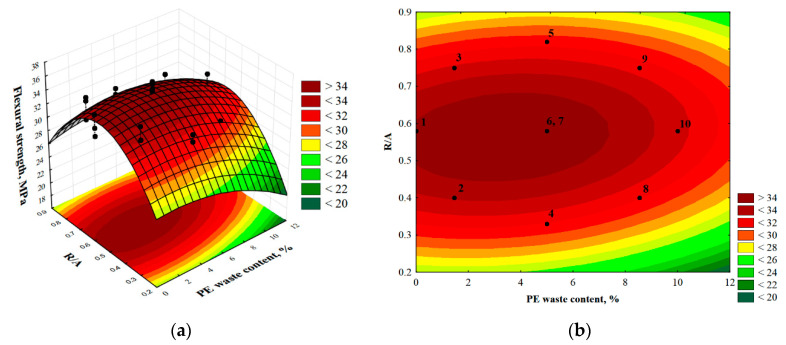
Three-dimensional (**a**) and contour (**b**) plot of the response surface for flexural strength.

We can see that the value of the fc function depends linearly on the percentage of PE waste content and its influence is small as evidenced by the −0.06 coefficient. On the other hand, the influence of the second input variable, the R/A ratio, on the values of the f_f_ function is described by a quadratic relationship, but in this case the influence of the quadratic factor is small because the values of the R/A ratio are fractional. The spatial and contour plot of this function is shown in Figure 12. The shape of these plots confirms that the flexural strength is significantly influenced by both input variables, namely the PE waste content and the resin/aggregate ratio. The function has a maximum, which is located in the vicinity of the predefined center of the experimental plan, marked as points numbered 6 and 7 (the composite samples corresponding to these plan points are characterized by 5% PE waste content and R/A equal to 0.58). This confirms that the range of variation of the input data was correctly selected.

### 3.2. Compressive Strength

**Figure 13:**
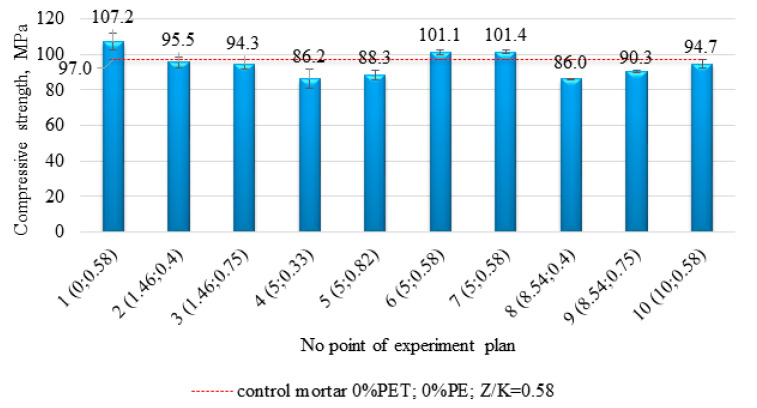
The compilation of the mean compressive strength values for the samples corresponding to each point of the experimental plan.

**Figure 14:**
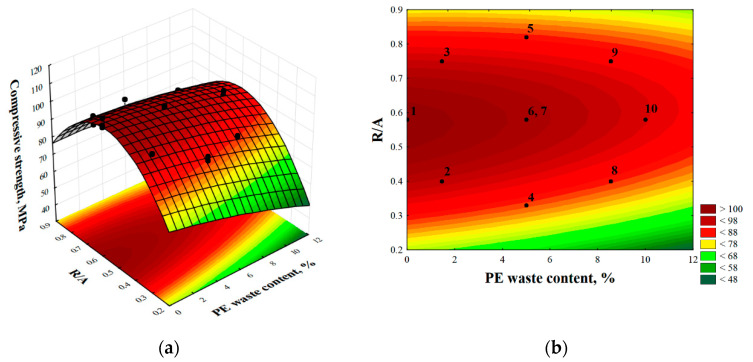
Three-dimensional (**a**) and contour (**b**) plot of the response surface for compressive strength.

Mean values of compressive strength and standard deviations of these parameters calculated for each composition are shown in Figure 13. The highest results of 107.2 MPa, 101.4 MPa and 94.7 MPa were obtained for samples with 0%, 5%, and 10% of PE waste content, respectively, at R/A ratio of 0.58. Three-dimensional and contour plots of the approximation function (Figure 14) confirm the fact of decreasing compressive strength values with increasing amount of PE waste added. However, even with 10% substitution of sand by PE waste (10 point of the experiment plan), the obtained strength value, equal to 94.7 MPa, is still at a very high level and differs from the result obtained for the control sample (the value of 97.0 MPa corresponding to the control sample is marked in Figure 13 with a horizontal, dashed line) only by 2.3 MPa. All the compressive strength values determined for the test composite samples are significantly higher than those obtained by Reis et al. for epoxy mortars modified with shredded PET waste [29]. Despite the high degree of crystallinity and roughness of PE particles, they are more susceptible to compressive loading than quartz sand, which may be the reason for the decrease in compressive strength.

The response surface function of general Formula (1) adjusted to the results of compressive strength measurements took the form of (3):

f_c_ = 25.34 − 1.77(%PE) + 284.51(R/A) − 253.1(R/A)^2^ + 2.19(%PE)(R/A) (3)

Comparing this formula with relation (2) describing the flexural strength, one can notice that there is an additional interaction component, since the last component of the sum contains the product of the %PE waste content and the R/A ratio, and the resulting value of the fc function depends on it. If one of these parameters increases and the other one decreases, the influence of this component on the final value of the compressive strength is minor.

### 3.3. Bulk Density

**Figure 15:**
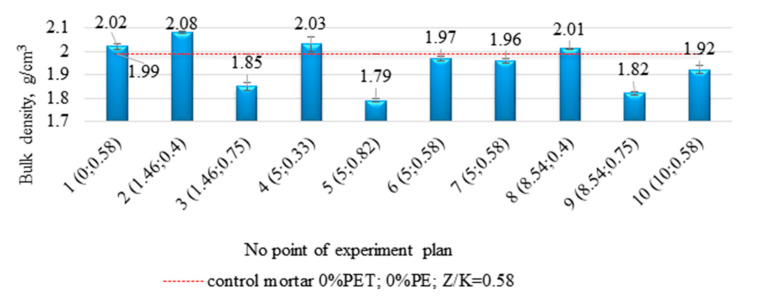
The compilation of mean volumetric density values calculated for each point of the experimental plan.

The mean volumetric density values along with the standard deviation calculated at each point in the experimental plan are summarized in Figure 15.

**Figure 16:**
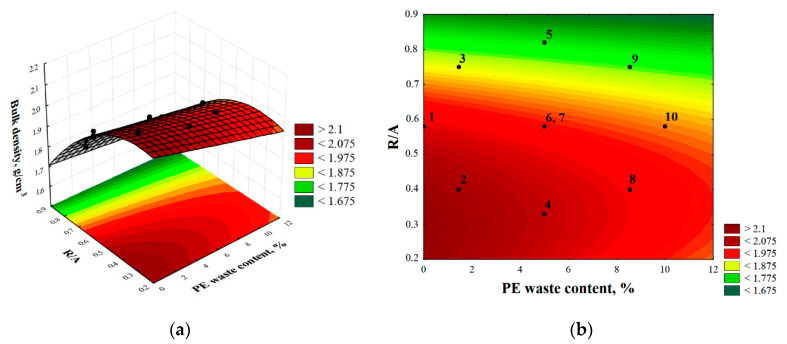
Three-dimensional (**a**) and contour (**b**) plot of response surface for bulk density.

It can be seen from Figure 16a,b that an increase in the proportion of resin and PE waste content in the epoxy composites reduces the bulk density of these materials.

The volumetric density values ranged from 1.79 g/cm^3^ to 2.08 g/cm^3^. The response surface function of the general Formula (1) adjusted to the volumetric density results took the form of (4):

d_b_ = 2.02 − 0.02(%PE) + 0.59(R/A) − 1.04(R/A)^2^ (4)

Analyzing the parameters of this equation, again for this feature, it can be seen that the volumetric density value of the samples is more influenced by the resin to aggregate ratio (R/A) than by the degree of substitution of sand by PE waste (coefficient −0.02).

### 3.4. Multiple Output Optimization: A Response Utility Profile

**Figure 17:**
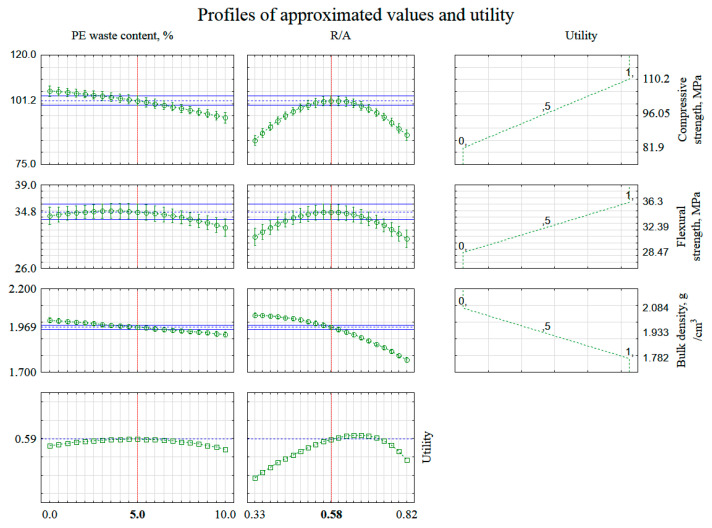
Profiles of approximated values and utility for the method of general function optimization.

Using the utility profile module of the Statistica program, optimization of all three designated mortar properties (output quantities) was performed for the model under consideration. Resin mortars should be durable and at the same time relatively light, i.e., with a low bulk density. Mortar composition should be chosen in such a way as to optimize the overall utility of the composite. For the obtained second-order response surface models, the program calculated the values of the input quantities corresponding to the minimum and maximum values of the given surface (i.e., the critical values of the given surface together with the corresponding eigenvalues and eigenvectors thus describing the curvature and orientation of the response surface). For the three properties marked (functions: f_f_, f_c_, d_b_), a utility function was defined that reflects the most desirable values of the output quantities and the weight of each of these quantities for the total utility. This approach made it possible to plot the profiles of the utility function (calculated from the values of the approximated output quantities) by assigning to each possible value of the attribute under study a value in the interval [0, 1] indicating satisfaction with the outcome at that level. A utility of 0.0 (undesirable) was assigned to approximate values for flexural strength below 28.47 MPa, compressive strength below 81.9 MPa, and bulk density above 2.084 g/cm^3^, respectively. In turn, the utility of 1.0 (highly desirable) was assigned to approximate values of flexural strength above 36.3 MPa, compressive strength above 110.2 MPa, and a bulk density below 1.782 g/cm^3^. Whereas the utility increasing linearly 0.0–1.0 was defined for flexural strength from 28.47 MPa to 36.3 MPa, compressive strength from 81.9 MPa to 110.2 MPa and for decreasing values of the volume density function from the range 2.084–1.782 g/cm^3^. The search for the composition of the mortar that would provide the most desirable composite properties was based on the general optimization of functions. The results are shown in Figure 17.

**Figure 18:**
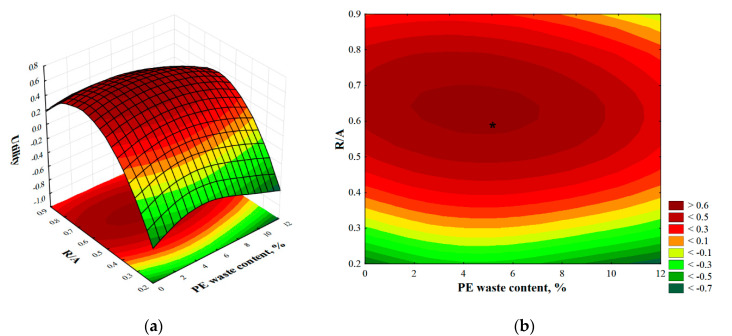
Three-dimensional (**a**) and contour (**b**) plot of total utility.

Optimum value of flexural and compressive strength and volumetric density were obtained for the percentage of PE waste addition at 5 vol.% and resin to aggregate ratio (R/A) equals 0.58. The values of compressive strength, flexural strength, and volumetric density at the point of maximum utility are 101.2 MPa, 34.8 MPa, and 1.969 g/cm^3^, respectively. The total utility index reached 0.59, confirming the relatively close location of critical points for all three output variables. The spatial and contour plot of total utility is shown in Figure 18.

The *-marked point in Figure 18b, indicating the centrality of the utility index in the area described by the input variables, confirms that the experimental plan was correctly defined.

## 4. Conclusions

Based on the research, it can be concluded that:

- Epoxy mortars can be successfully modified with such plastic wastes as poly(ethylene terephthalate) and polyethylene.
- Both the addition of the glycolysate obtained on the basis of PET waste and the agglomerate of PE waste influence the plasticity of the epoxy mortars obtained and improve the flexural strength.
- Flexural strength of the mortars at 5% substitution of sand with PE waste increased by 6.6% and at 10% substitution it was comparable with the values obtained for the mortars without the waste additive and amounted to 33.3 MPa.
- The addition of PE waste agglomerate slightly decreased the compressive strength of epoxy mortars, but even at 10% substitution of sand with PE waste the strength remained at a very high level of 94.7 MPa.
- Applying the multiple output optimization, it was shown that the most advantageous values of strength parameters and bulk density could be simultaneously obtained for mortars characterized by resin to aggregate ratio (R/A) equaling 0.58 and PE waste content at the level of 5 vol.%.
- Environmental concerns cast a shadow over the production, use, and consumption of the plastic. The proposed modification of epoxy mortars with plastic waste may be a way to solve them in accordance with the principles of modern, low-carbon, resource-, and energy-efficient economy, and lead to the implementation of goals adopted in the plans of sustainable development of EU countries by 2030.

## Figures and Tables

**Table 1:** The composition of the mortar for individual points of the experiment plan.

Experiment Plan Points	1	2	3	4	5	6	7	8	9	10
PE waste content, % vol.	0.0	1.46	1.46	5.0	5.0	5.0	5.0	8.54	8.54	10.0
Ratio of volumes of resin to aggregate R/A	0.58	0.40	0.75	0.33	0.82	0.58	0.58	0.4	0.75	0.58

**Table 2:** Chemical composition of the quartz sand.

Chemical Composition	SiO_2_	CaO	MgO	Al_2_O_3_	K_2_O	Others
%	87.14	1.30	0.38	7.80	1.65	1.73

## Data Availability

Not applicable.
